# Supplementary material for: The HslV Protease from Leishmania major and Its Activation by C-terminal HslU Peptides
Source: Int J Mol Sci. 2019 Feb 26;20(5):1021. doi: 10.3390/ijms20051021 (PMC6429459; doi:10.3390/ijms20051021)
Supplement: Supplementary file 1 [file ijms-20-01021-s001.pdf]

Kebe et al., supplementary information

Abbreviations. *Aloc*, allyloxycarbonyl; *AMC*, 7-Amino-4-methylcoumarin; *DCM*, dichloromethane; *DIEA*, diisopropylethylamine; *DMF*, dimethylformamide; *DMSO*, dimethylsulfoxide; *Fmoc*, fluorenylmethyloxycarbonyl; *HBTU*, *N*-[(1*H*-benzotriazol-1-yloxy) (dimethylamino)methylene]- *N*-methylmethanaminium hexafluorophosphate; *HPLC*, high performance liquid chromatography; *LC/MS*, liquid chromatography coupled to mass spectrometry; *NMP*, *N*-methyl-pyrrolidone; *O2Oc*, 8-amino-3,6-dioxaoctanoyl; *PS*, polystyrene; *TFA*, trifluoroacetic acid; *TFE*, trifluoroethanol; *TIS*, Triisopropylsilane; *TNBS*, trinitrobenzenesulfonic acid.

Supplementary experimental procedures

Peptide synthesis.

Peptide chain assembly was stepwise accomplished following an Fmoc/tBu strategy. It was either performed manually in a plastic syringe equipped with frit or with the help of a microwave-assisted automatic peptide synthesizer from CEM. Fmoc-protected amino acid derivatives, HBTU, Wang-polystyrene resin loaded either with Fmoc-Leu-OH (0.6 mmol/g) or Fmoc-Ala-OH (0.6 mmol/g), and 2-chloro-trityl chloride polystyrene resins (100-200 mesh, 1.6 mmol/g) were purchased from IrisBiotech or Novabiochem. Other reagents and solvents were purchased from Acros Organics, Alfa Aesar, Carlo Erba or Sigma-Aldrich and used without further purification. Solvents used for HPLC and LC/MS were of HPLC grade. All final compounds were purified by reversed-phase HPLC and the purity assessed by analytical reversed-phase HPLC and LC/MS was found superior to 95 %.

1. Anchoring of the C-terminal residue on 2-chlorotrityl chloride-PS resin. The resin was conditioned for 20 min in dry DCM/DMF (50:50). After filtration, the first residue (3 equiv.) was loaded onto the resin in suspension in dry DMF/DCM (50:50) in the presence of DIEA (4.5 equiv.). After 15h stirring at room temperature, the resin was filtered, washed with DCM and dried. The loading was determined on small portions (3-5 mg exactly weighed) of resin, which were treated with a 50:50 solution of piperidine in DCM to remove the Fmoc protecting group. The resulting dibenzofulvene-piperidine adduct was quantified by its absorbance at 301 nm (ε_M = 7800 M^{-1}).

2. Manual synthesis. The resin (either 2-chlorotrityl or Wang) loaded with the first Fmoc-amino acid residue (Fmoc-Leu-OH or Fmoc-Ala-OH or Fmoc-D-Leu-OH) was first conditioned for 15 min in DMF. The Fmoc-protected amino acids were then added by a succession of deprotection and coupling steps. Fmoc removal was performed through two consecutive treatments with piperidine/DMF (20/80) for 10 and 25 min. Coupling of Fmoc-protected amino acids (3 equiv. according to the resin loading) was carried out in DMF or NMP (1-methyl-2-pyrrolidone) in the presence of HBTU (3 equiv.) and DIEA (4 equiv.) for 3 h. After each step, the resin was washed with DMF, MeOH and DCM. Monitoring of the deprotection and coupling steps was performed using the TNBS (trinitrobenzenesulfonic acid) test. A second coupling was performed when necessary.

TNBS test: after each coupling and deprotection step, an aliquot portion of resin beads was collected and treated with one drop of a 1 M TNBS solution in DMF and one drop of a 10% DIEA solution in DMF (v/v). Red beads after 1 min at room temperature indicated the presence of free amine groups.

At intermediate steps the on-resin growing peptide was checked by cleaving 2-3 mg of resin with 500 µL of the below-defined TFA mixture and the released crude product was analyzed by HPLC and LC-MS.

3. Automatic microwave-assisted peptide synthesis.

Peptide synthesis was performed using an automated peptide synthesizer CEM Liberty. Each synthesis was performed on a 0.15 mmol scale, using 2-chlorotrityl polystyrene resin pre-loaded either with Fmoc-Leu-OH or Fmoc-Ala-OH (0.6 mmol/g). The Fmoc protected amino acids (0.2 M), the coupling reagent HATU (0.5 M) and DIEA (2 M) in NMP were added to the vessel and the mixture was stirred using nitrogen flux under microwave irradiation (40 W) at 70 °C for 300 seconds. After each coupling reaction, resin was submitted to two consecutive deprotection cycles. First with 20 mL of DMF/piperidine 80/20 v/v solution under microwave irradiation (40 W) at 75°C for 30 seconds and the second under the same conditions during 180 seconds. In the case of Asp(OtBu) and Glu(OtBu) residues, Fmoc removal was performed without heating (two steps of 5 and 20 min) to avoid epimerization. Double coupling was performed for Fmoc-Arg(Pbf)-OH.

4. Cleavage of peptide resins. The peptides were simultaneously cleaved from the resin and deprotected by 2h treatment with a mixture of trifluoroacetic acid, triisopropylsilane and water (95:2.5:2.5). After removal of the resin by filtration, the TFA was concentrated in vacuo. Compounds were precipitated by addition of diethyl ether and recovered after centrifugation. The pellet was washed twice with diethyl ether. All compounds were obtained with an average yield of 20%. They were purified by reverse-phase HPLC. All compounds were above 90-95% purity.

5. Analysis and purification. RP-HPLC analyses were performed using a Waters Alliance 2690 HPLC by loading the sample in solution in acetonitrile/water (50/50 v/v) mixture onto a Chromolith SpeedRod C18 column (0.46 x 5 cm), and applying a linear gradient (0-100%) of eluent B in A over 5 min (flow rate: 3 mL/min). Detection was made at λ = 214 nm. Eluent A: 0.1% aqueous TFA; eluent B: acetonitrile/0.1% TFA.

Purification by preparative RP-HPLC was performed on a Waters Delta Pak C18 column (40 x 100 mm, 15 µm, 100 Å) by elution with a linear gradient of eluent B in A at a 1%/min rate (flow rate: 28 mL/min). Eluent A: 0.1% aqueous TFA; eluent B: acetonitrile/0.1% TFA.

Mass spectrometry: samples were prepared in acetonitrile/water (50/50 v/v) mixture. The LC-MS system consisted of a Waters Alliance 2690 HPLC, coupled to a Waters-Micromass ZQ spectrometer (electrospray ionization mode, ESI+). All analyses were carried out using a RP C18 monolithic Onyx Phenomenex column (25 x 4.6 mm), linear gradient (0–100%) of eluent B in A over 3 min (flow rate: 3 mL/min). Eluent A: 0.1% aqueous formic acid; eluent B: acetonitrile/0.1% formic acid. Positive ion electrospray mass spectra were acquired at a solvent flow rate of 100–500 µL/min. Nitrogen was used as both the nebulizing and drying gas. The data were obtained in a scan mode in 0.1 s intervals; 10 scans were summed up to get the final spectrum.

The HPLC and LC-MS retention times, calculated and measured molecular masses of the peptides are reported in **Table 1**.

6. Synthesis of cyclic peptides.

Cyclic peptides with a lactam bridge (**37-43**) formed between the side-chains of a L or D aspartyl residue and that of a diamino acid residue (Lys, lys, Orn, Dap) were prepared by introducing these residues as their allyl and aloc side-chain-protected derivatives, respectively. After peptide assembly, the allyl and aloc groups were removed by treatment of the resin with $Pd[PPh_3]_4$ (0.2 equiv.) and phenylsilane (24 equiv.) in anhydrous DCM. After 4 h stirring at r.t. with frequent degassing, the resin was filtered and washed twice with DCM, DMF, DCM and the treatment was repeated. Cyclisation was then performed on the solid phase by coupling the free carboxylic and amine groups in the presence of HBTU (1.5 equiv.) and DIEA (2.5 equiv.) in NMP for 4h. After washings with DMF and DCM, the completion of the reaction was assessed by the TNBS test.

7. Synthesis of dimer. Dimers of LmHslU2 C-terminal octapeptide **5** and dodecapeptide **12** were prepared as follows.

a) Synthesis of the monomers: the octa- and dodecapeptide extended on their N-terminus by three O2Oc residues were first assembled on a 2-chlorotrityl resin as described above. Each peptide-resin was then separated in two equal portions, onto which was coupled either pentynoic acid or 3-azidopropionic acid (see synthesis below). Full cleavage using TFA/TIS/H_2O yielded the following deprotected peptides, which were used for the click reaction without further purification:

A = Pentynoyl-$(O2Oc)_3$-Val-Asn-Leu-Ala-Lys-Tyr-Leu-Leu-OH: LC-MS analysis: t_R = 1.28 min; 760.5 ($[M+2H]^{2+}$), 507.5 ($[M+3H]^{3+}$).

B = 3-Azidopropionyl-$(O2Oc)_3$-Val-Asn-Leu-Ala-Lys-Tyr-Leu-Leu-OH: LC-MS analysis: t_R = 1.29 min; 769.1 ($[M+2H]^{2+}$), 513.2 ($[M+3H]^{3+}$).

C = Pentynoyl-$(O2Oc)_3$-Leu-Gln-Lys-Asn-Val-Asn-Leu-Ala-Lys-Tyr-Leu-Leu-OH: LC-MS analysis: t_R = 1.18 min; 967.1 ($[M+2H]^{2+}$), 644.8 ($[M+3H]^{3+}$).

D = Azidopropionyl-$(O2Oc)_3$-Leu-Gln-.Lys-Asn-Val-Asn-Leu-Ala-Lys-Tyr-Leu-Leu-OH: LC-MS analysis: t_R = 1.17 min; 975.5 ($[M+2H]^{2+}$), 650.5 ($[M+3H]^{3+}$).

b) Synthesis of 3-azido-propionic acid: the synthesis of 3-azidopropionic acid was adapted from (1). To a solution of 3-bromopropionic acid (3.0 g, 19.6 mmol) in acetonitrile was added sodium azide (1.9 g, 29.2 mmol). The solution was refluxed for 3h and then diluted with DCM 40 mL. The organic phase was washed twice with 0.1 N HCl, then the aqueous phases were extracted with DCM (2 times) and EtOAc (2 times). The organic phases were mixed, dried over Na_2SO_4, filtered and evaporated to give an orange oil, which was purified on a silica gel column (DCM/AcOH, 99:1) to yield a colorless oil (964 mg, 42.8%). R_f (DCM/AcOH, 99:1) = 0.19; IR: ν (cm^{-1}) 2094, 1708; ^1H NMR (300 MHz,

$CDCl_3$), δ (ppm), 2.64 (t, J = 6.5 Hz, 2H), 3.59 (t, J = 6.5 Hz, 2H), 8.28 (br, 1H); ^{13}C NMR (75 MHz, $CDCl_3$), δ (ppm), 176.6, 46.8, 34.0; ESI^- LC-MS analysis: t_R = 0.45 min; 114.1 ($[M-H]^-$).

c) Synthesis of dimers by Copper(I)-Catalyzed Azide-Alkyne cycloaddition, (CuAAC, "Click" chemistry).

- Dimer of octapeptide **5**: compounds A (25 mg, 16.2 μmol) and B (25 mg, 16.2 μmol) were solubilized in PBS (PBS from Lonza, BE17-516-F) with 10% EtOH (25mL). Were then added a 0.2 M aqueous solution of $CuSO_4,5H_2O$ (68 μL, 13.6 μmol) and a 0.6 M aqueous solution of sodium ascorbate (68 μL, 40.8 μmol). The reaction was followed by reverse phase HPLC analysis. After 3h, the medium was acidified with 100 μL of TFA and directly loaded onto a preparative HPLC for purification to yield 19.3 mg of purified dimer. LC-MS analysis: t_R = 1.31 min; 765.1 ($[M+4H]^{4+}$), 612.2 ($[M+5H]^{5+}$).

- Dimer of dodecapeptide **12**: compounds C (50 mg, 23 μmol) and D (50 mg, 23 μmol) were solubilized in a 50:50 mixture of PBS (PBS from Lonza, BE17-516-F) and tBuOH (25 mL). Were then added a 0.2 M aqueous solution of $CuSO_4,5H_2O$ (97 μL, 19.4 μmol) and a 0.6 M aqueous solution of sodium ascorbate (97 μL, 68.2 μmol). The reaction was followed by reverse phase HPLC analysis. After 15h, the medium was acidified with 100 μL of TFA and directly loaded onto a preparative HPLC for purification to yield 4 mg of purified dimer. LC-MS analysis: t_R = 1.23 min; 971.1 ($[M+4H]^{4+}$), 777.1 ($[M+5H]^{5+}$).

8. Synthesis of the fluorogenic substrate, Z-Glu-Val-Asn-Leu-AMC. The substrate was obtained by condensing the two fragments Z-Glu(OtBu)-Val-Asn(Trt)-OH and H-Leu-AMC, TFA salt.

a) Z-Glu(OtBu)-Val-Asn(Trt)-OH: the peptide was synthesized on a 2-chlorotrityl resin following a Fmoc strategy as described above. After assembly, the fully protected peptide was obtained by cleaving the resin with the mixture DCM/TFE/AcOH (7:2:1) three times (1h and twice 10 min). The solvents were evaporated under vacuum and the peptide was precipitated in water, filtered, dried and used in the next step without purification (95% pure). LC-MS analysis: t_R = 2.08 min; 793.3 ($[M+H]^+$).

b) Boc-Leu-AMC: to a solution of Boc-Leu-OH, H_2O (232 mg, 0.93 mmol) and pyridine (70 μL) in dioxane (3.6 mL) were added $(Boc)_2O$ (249 mg, 1.14 mmol) and AMC (200 mg, 1.14 mmol) and the mixture was stirred for 24h at room temperature. After dilution with 20 mL AcOEt, the organic phase was washed with water, saturated aqueous $NaHCO_3$ (3 times), water, aqueous 1M $KHSO_4$ (3 times), water and brine. The organic phase was dried on Na_2SO_4, filtered and evaporated. The residue was purified on a silica gel column (hexane/AcOEt, 72:28) to yield a white solid (200 mg, 55.4%). LC-MS analysis: t_R = 1.80 min; 389.2 ($[M+H]^+$).

c) H-Leu-AMC, TFA: Boc-Leu-AMC was treated with TFA/DCM (50:50) for 30 min at room temperature. The solution was evaporated and the oily residue precipitated in diethyl ether. After centrifugation, the pellet was washed twice with ether and dried to yield a white solid, which was used in the next step without further purification (175 mg, 84.5%, > 95% pure). LC-MS analysis: t_R = 1.10 min; 289.2 ($[M+H]^+$).

d) Z-Glu-Val-Asn-Leu-AMC: to a solution of Z-Glu(OtBu)-Val-Asn(Trt)-OH (345 mg, 0.43 mmol) and H-Leu-AMC, TFA (175 mg, 0.43 mmol) in dry DMF (9 mL) were added at 4°C TEA (61 μL, 0.43 mmol), DIEA (151 μL, 0.87 mmol) and BOP (231 mg, 0.52 mmol). After 3h stirring, the solvent was eliminated under vacuum and the oily residue was precipitated in water. The precipitated was recovered by filtration and dried. The protecting groups were removed by treatment with $TFA/TIS/H_2O$ as described above. The substrate was finally purified by reverse phase HPLC to yield a white powder (60 mg, 15.2%). LC-MS analysis: t_R = 1.55 min; 765.3 ($[M+H]^+$).

CD experiments.

CD spectra (Figure S2) were recorded on a JASCO J-815 spectropolarimeter from 190 to 260 nm (data pitch of 0.1 nm, continuous scanning mode, 200 nm/min, 3 scans) at 20 °C, using a 1 mm path length cuvette. Baseline was corrected by subtracting the background from the sample spectrum. Peptides were solubilised in 50 mM Tris-HCl, pH 8.0 buffer at an estimated concentration of 400 μM according to their apparent molecular mass (i.e. M + xTFA with x = number of basic groups). The solutions were then diluted twice (25 mM Tris and around 200 μM peptides final concentrations) with: water: 0% TFE; water/TFE (4:1): 10% TFE; water/TFE (1:1): 25% TFE; TFE: 50% TFE; and transferred to a 200 μL cuvette.

The final peptide concentrations were determined by measuring the absorbance at $\lambda = 276$ nm and taking a molar absorption coefficient ε of 1450 for all peptides containing one tyrosine. They are reported in **Table S1**. These values were used to calculate the per residue molar ellipticities $[\theta] = \theta/(10 \times c_r \times l)$, where c_r is the mean residue molar concentration and l is the path length. Percent helicities were calculated from θ values at $\lambda = 222$ nm according to (2).

References

1. Grandjean, C., Boutonnier, A., Guerreiro, C., Fournier, J.-M., and Mulard, L. A. (2005) On the preparation of carbohydrate-protein conjugates using the traceless Staudinger ligation. *J. Org. Chem.* **70**, 7123–7132
2. Greenfield, N., and Fasman, G. D. (1969) Computed circular dichroism spectra for the evaluation of protein conformation. *Biochemistry*. **8**, 4108–4116
3. Sung, K. H., Lee, S. Y., and Song, H. K. (2013) Structural and Biochemical Analyses of the Eukaryotic Heat Shock Locus V (HslV) from Trypanosoma brucei. *J. Biol. Chem.* **288**, 23234–23243

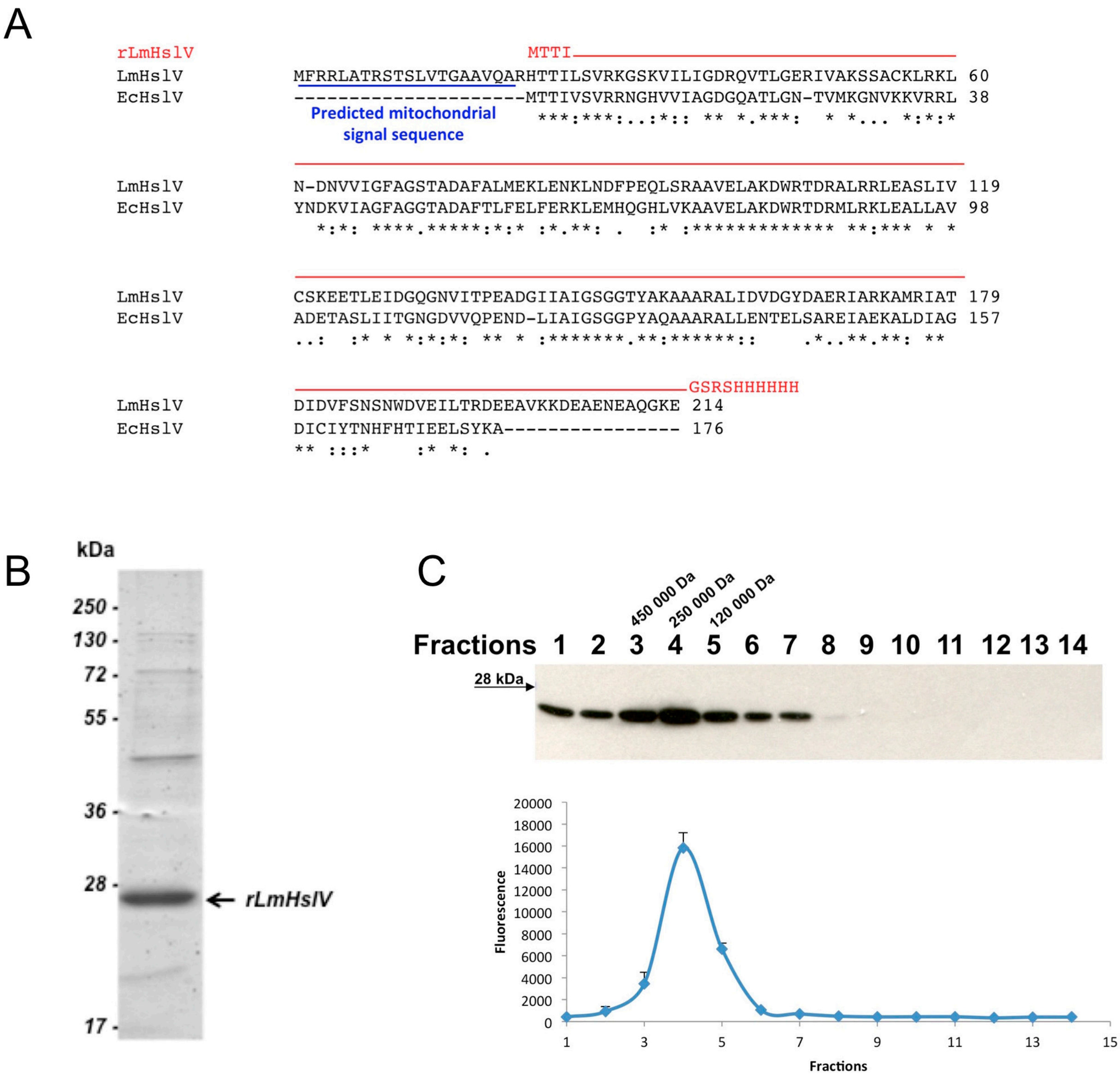

Figure S1. Characterisation of recombinant LmHslV. (A) Alignment of the sequences of *L. major* HslV (LmHslV), recombinant LmHslV (rLmHslV) and *E. coli* HslV (EcHslV). The predicted mitochondrial signal sequence of LmHslV, underlined in blue, has been removed in rLmHslV (red). (B) After Ni^{2+}-NTA purification, rLmHslV was analyzed by 13% SDS-PAGE and subsequent coomassie blue staining. rLmHSLV monomer is indicated. (C) *Gel filtration analysis of rLmHslV:* 10µg of LmHslV were separated by gel filtration (Superose 12 PC 3.2/30) in buffer A (see "experimental procedures"). 15 fractions of 100µL were collected. 20µl of each fraction were resolved by 13% SDS-PAGE and immunoprobed with anti-6His antibodies (top panel). In parallel, 15µl of each fraction were incubated for 20 min at 37°C in the presence of 100µM of the substrate Z-EVNL-AMC and 500µM of the activating peptide LmC12-U2, **10**, in a final volume of 50µl (bottom panel).

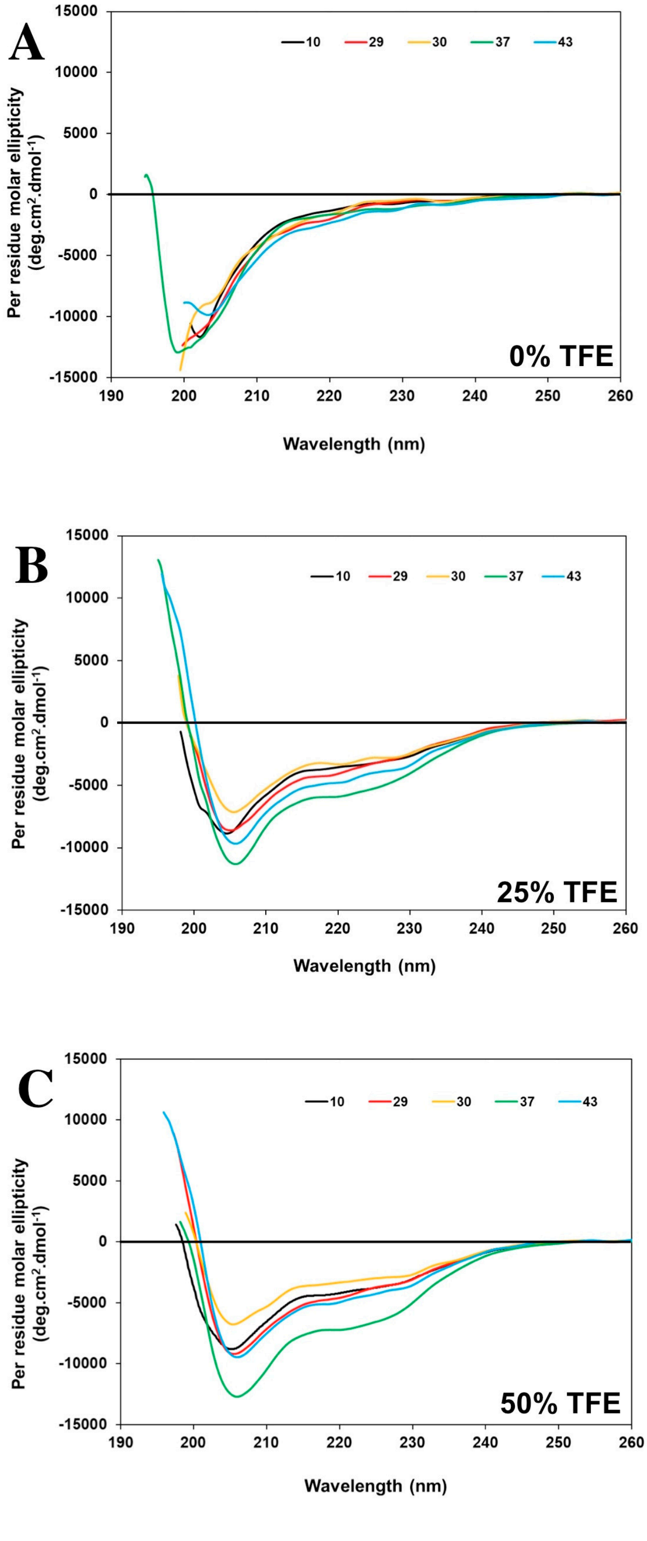

Figure S2. Superimposition of CD spectra of LmC12-U2 analogues **10**, **29**, **30**, **37**, **43**, in 25 mM Tris-HCl, pH 8.0 buffer containing: A) 0% TFE; B) 25% TFE; C) 50% TFE. See table S2 for peptide concentrations in these experiments.

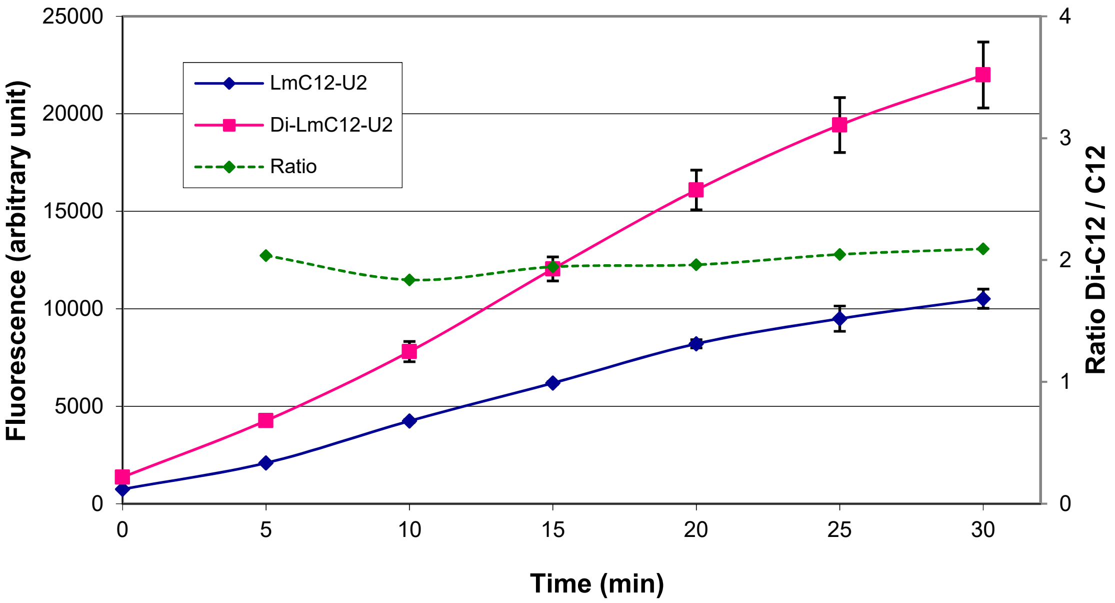

Figure S3. *Effect of LmC12-U2 dimerization*. LmHslV has been incubated at 37°C for 30 min with 550µM of the peptide **10** (LmC12-U2) or of its dimer form (Di-LmC12-U2) (**12**), in the presence of the substrate Z-EVNL-AMC (100µM). The fluorescence generated by the cleavage of the substrate was recorded at 5 min intervals. The dotted line represents the ratio at each time point between the fluorescence values obtained with the two peptides.

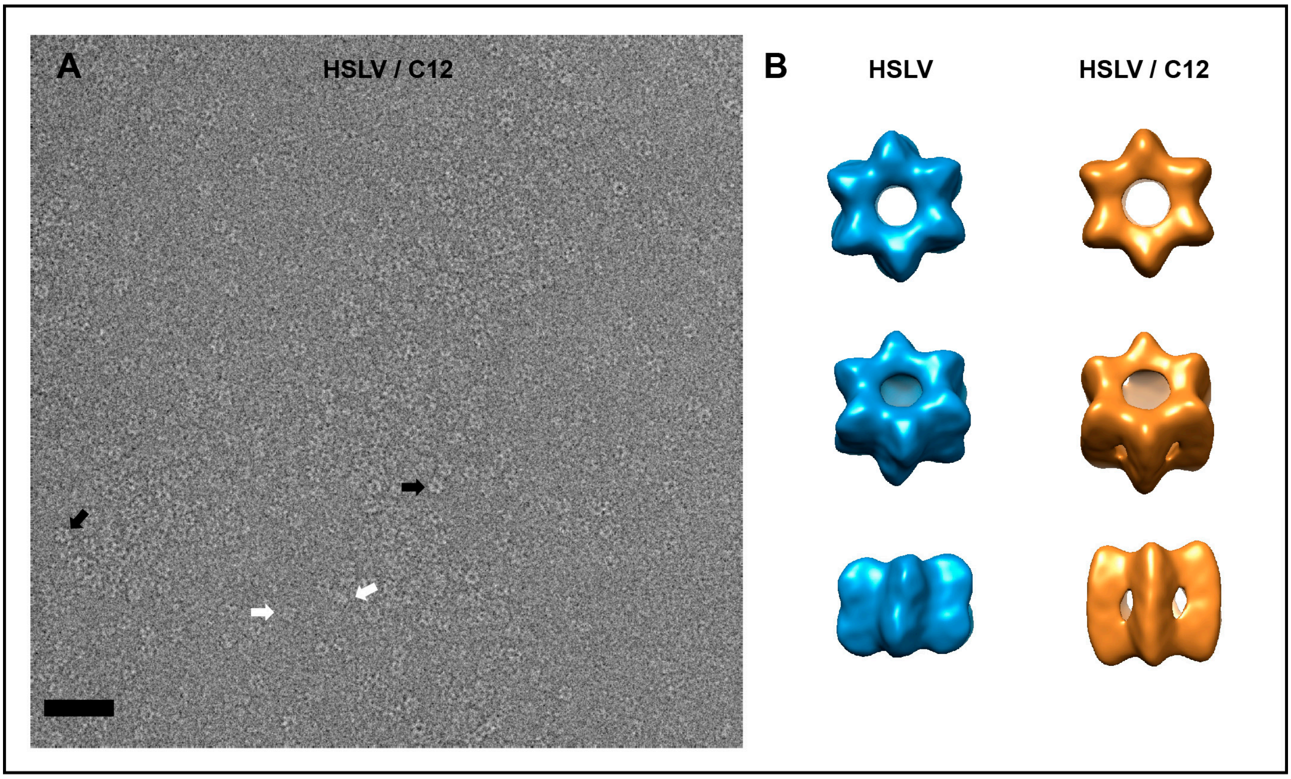

Figure S4. *Cryo-EM visualization of LmHslV and LmHslV-LmC12-U2***.** (A) Field of frozen-hydrated HslV-C12-U2 particles. Black arrows point to top views presenting the star-like shape while the white arrows show side views. Scale bar, 50 nm. (B) 3D reconstruction of HslV and HslV-C12-U2, respectively. A 90° rotation was imposed to go from the top view to the bottom view of the complexes.

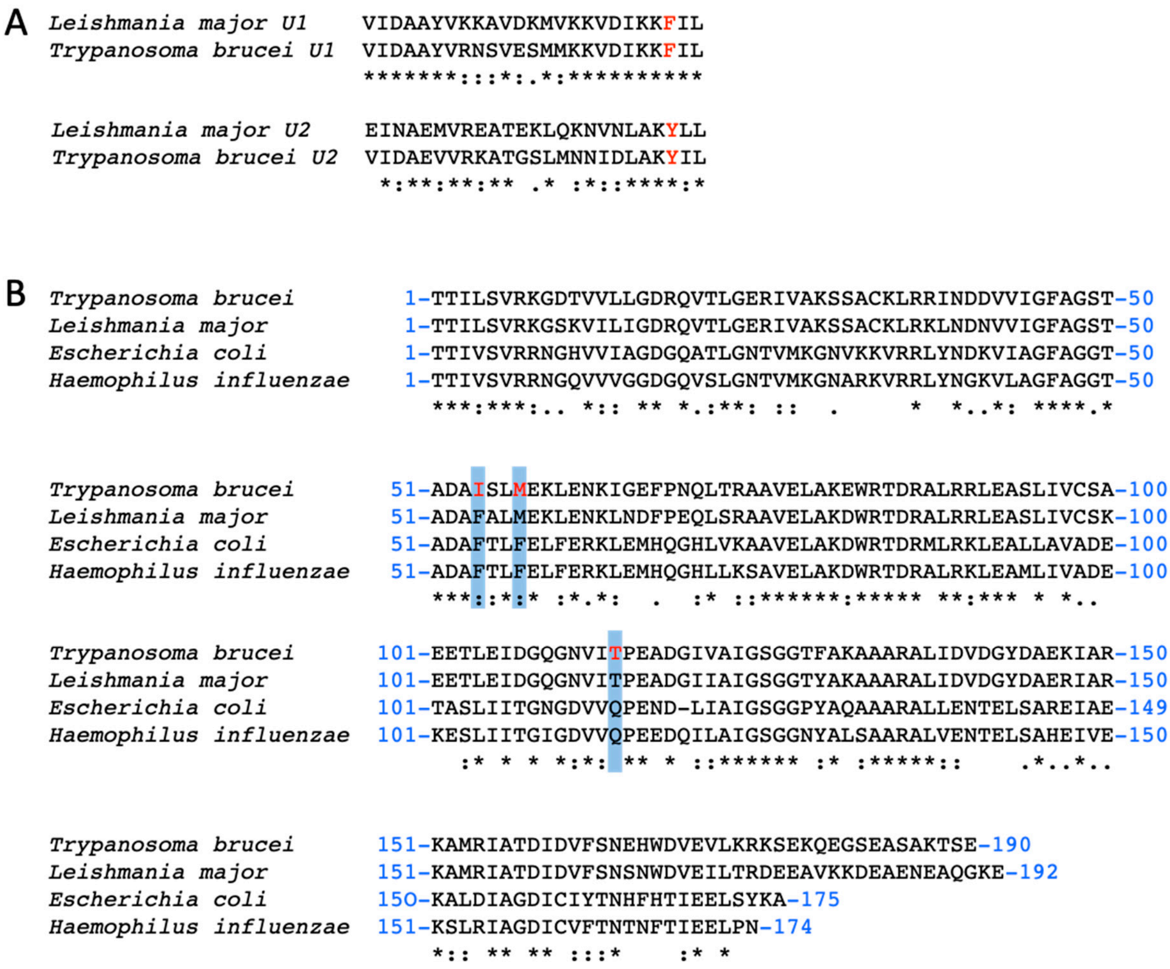

Figure S5. *Sequence alignments of (A) the C-terminal ends of HslU1 and HslU2 from Trypanosoma brucei* and *Leishmania major* and (B) *HslV from Trypanosoma brucei, Escherichia coli , Leishmania major and Haemophilus influenzae.* In (A), the antepenultimate phenylalanine (F) of TbU1 and LmU1 and tyrosine (Y) of TbU2 and LmU2 which are discussed in the main text are highlighted in red. In (B), residues are numbered as in the mature proteins (starting with the N-terminal TTI motif). In red are the three residues of TbHslV described in Sung et al. (3) as important for accommodating the C-terminal tail of HslU (see discussion of the main text). The blue boxes underline the corresponding residues in *L. major*, *E. coli* and *H. influenzae* HslVs.
UniProt IDs of the sequences : EcHslV, P0A7B8 ; HiHslV, P43772 ; LmHslV, Q4Q116 ; TbHslV, Q383Q5 ; LmHslU1, Q4QFH5 ; TbHslU1, Q57VB1 ; LmHslU2, Q4QI03 ; TbHlsU2, Q382V8.

Table S1. HslU C-ter peptide analogues.

Cpd	Structure	M Calc.[a]	m/z Obs. ions[b]	t_R min (LC-MS)[c]
C-ter HslU: Octamers				
LmHslU1				
**1** H-LmC8-U1	H-Val-Asp-Ile-Lys-Lys-Phe-Ile-Leu-OH	974.6 (975.2)	326.1 (3+) 488.4 (2+) 975.8 (1+)	1.19[d] (1.06)
**2** Ac-LmC8-U1	Ac-Val-Asp-Ile-Lys-Lys-Phe-Ile-Leu-OH	1016.6 (1017.2)	509.5 (2+) 1017.8 (1+)	1.31[d] (1.17)
LmHslU2				
**3** H-LmC8-U2	H-Val-Asn-Leu-Ala-Lys-Tyr-Leu-Leu-OH	932.6 (933.1)	467.4 (2+) 933.6 (1+)	1.25[d] (1.06)
**4** Ac-LmC8-U2	Ac-Val-Asn-Leu-Ala-Lys-Tyr-Leu-Leu-OH	974.6 (975.2)	488.4 (2+) 975.6 (1+)	1.42[d] (1.30)
**5** Dimer of LmC8-U2	1,2,3-Triazole-1,4=[CH_2-CH_2CO-(O2Oc)$_3$-Ala-Val-Asn-Leu-Ala-Lys-Tyr-Leu-Leu-OH]$_2$	3054.6 (3056.5)	612.2 (5+) 765.1 (4+) **3055.9**	1.58[d] (1.31)
EcHslU				
**6** H-EcC8-U	H-Glu-Asp-Leu-Ser-Arg-Phe-Ile-Leu-OH	991.5 (992.1)	497.0 (2+) 992.7 (1+)	1.23[d] (1.11)
**7** Ac-EcC8-U	Ac-Glu-Asp-Leu-Ser-Arg-Phe-Ile-Leu-OH	1033.5 (1034.2)	517.9 (2+) 1034.7 (1+)	1.32[d] (1.23)
C-ter HslU: Dodecamers				
LmHslU1				
**8** LmC12-U1	H-arg-O2Oc-Met-Val-Lys-Lys-Val-Asp-Ile-Lys-Lys-Phe-Ile-Leu-OH	1762.1 (1763.2)	353.6 (5+) 441.7 (4+) 588.7 (3+) 882.6 (2+) **1762.7**	1.81 (1.09)
**9** [Tyr[10]]-LmC12-U1	H-arg-O2Oc-Met-Val-Lys-Lys-Val-Asp-Ile-Lys-Lys-Tyr-Ile-Leu-OH	1778.1 (1779.2)	356.8 (5+) 445.6 (4+) 593.8 (3+) 890.0 (2+)	1.67 (0.99)
LmHslU2				
**10** LmC12-U2	H-arg-O2Oc-Leu-Gln-Lys-Asn-Val-Asn-Leu-Ala-Lys-Tyr-Leu-Leu-OH	1717.0 (1718.1)	430.4 (4+) 573.5 (3+) 859.5 (2+) **1717.6**	1.26[d] (1.00)
**11** [Phe[10]]-LmC12-U2	H-arg-O2Oc-Leu-Gln-Lys-Asn-Val-Asn-Leu-Ala-Lys-Phe-Leu-Leu-OH	1701.0 (1702.1)	426.4 (4+) 568.2 (3+) 851.5 (2+) **1701.4**	2.13 (0.97)
**12** Dimer of LmC12-U2	1,2,3-Triazole-1,4=[CH_2CH_2CO-(O2Oc)$_3$-Leu-Gln-Lys-Asn-Val-Asn-Leu-Ala-Lys-Tyr-Leu-Leu-OH]$_2$	3880.3 (3881.5)	647.9 (6+) 777.3 (5+) 971.3 (4+) 1294.7 (3+) **3881.2**	2.35 (1.21)
HslU from other origins				

**13**<br>TbC12-U2	H-arg-O2Oc-Leu-Met-Asn-Asn-Ile-Asp-Leu-Ala-Lys-Tyr-Ile-Leu-OH<br>(C12-U2 from *Trypanosoma brucei*)	1721.0<br>(1722.1)	575.0 (3+)<br>862.4 (2+)<br>**1722.4**	2.18<br>(1.25)
**14**<br>PfC12-U	H-arg-O2Oc-Phe-Ile-Lys-Gln-Tyr-Asp-Leu-Lys-Lys-Tyr-Ile-Ile-OH<br>(C12-U from *Plasmodium falciparum*)	1872.1<br>(1873.2)	469.3 (4+)<br>625.5 (3+)<br>937.6 (2+)<br>**1873.3**	1.85<br>(1.07)
**15**<br>EcC12-U	H-arg-O2Oc-Leu-Val-Ala-Asp-Glu-Asp-Leu-Ser-Arg-Phe-Ile-Leu-OH<br>(C12-U from *Escherichia coli*)	1690.9<br>(1691.9)	565.2 (3+)<br>847.2 (2+)<br>**1692.5**	2.10<br>(1.21)
**Ala-scan** (*C-ter LmHslU2*)				
**16**<br>[Ala^{5}]-LmC12-U2	H-arg-O2Oc-Leu-Gln-Lys-Asn-Ala-Asn-Leu-Ala-Lys-Tyr-Leu-Leu-OH	1689.0<br>(1690.0)	564.2 (3+)<br>845.5 (2+)<br>**1689.3**	1.41[d]<br>(0.97)
**17**<br>[Ala^{6}]-LmC12-U2	H-arg-O2Oc-Leu-Gln-Lys-Asn-Val-Ala-Leu-Ala-Lys-Tyr-Leu-Leu-OH	1674.0<br>(1675.0)	559.4 (3+)<br>838.6 (2+)<br>**1675.2**	1.48[d]<br>(1.00)
**18**<br>[Ala^{7}]-LmC12-U2	H-arg-O2Oc-Leu-Gln-Lys-Asn-Val-Asn-Ala-Ala-Lys-Tyr-Leu-Leu-OH	1675.0<br>(1676.0)	420.0 (4+)<br>559.4 (3+)<br>838.7 (2+)<br>**1675.5**	1.33[d]<br>(0.91)
**19**<br>[Ala^{9}]-LmC12-U2	H-arg-O2Oc-Leu-Gln-Lys-Asn-Val-Asn-Leu-Ala-Ala-Tyr-Leu-Leu-OH	1660.0<br>(1661.0)	554.5 (3+)<br>831.1 (2+)<br>**1660.3**	1.46[d]<br>(1.08)
**20**<br>[Ala^{10}]-LmC12-U2	H-arg-O2Oc-Leu-Gln-Lys-Asn-Val-Asn-Leu-Ala-Lys-Ala-Leu-Leu-OH	1625.0<br>(1626.0)	542.9 (3+)<br>813.6 (2+)<br>**1625.4**	1.43[d]<br>(0.96)
**21**<br>[Ala^{11}]-LmC12-U2	H-arg-O2Oc-Leu-Gln-Lys-Asn-Val-Asn-Leu-Ala-Lys-Tyr-Ala-Leu-OH	1675.0<br>(1676.0)	559.6 (3+)<br>838.8 (2+)<br>**1675.7**	1.32[d]<br>(0.93)
**22**<br>[Ala^{12}]-LmC12-U2	H-arg-O2Oc-Leu-Gln-Lys-Asn-Val-Asn-Leu-Ala-Lys-Tyr-Leu-Ala-OH	1675.0<br>(1676.0)	420.0 (4+)<br>559.5 (3+)<br>838.7 (2+)<br>**1675.6**	1.21[d]<br>(0.93)
***D*-scan** (*C-ter LmHslU2*)				
**23**<br>[leu^{7}]-LmC12-U2	H-arg-O2Oc-Leu-Gln-Lys-Asn-Val-Asn-leu-Ala-Lys-Tyr-Leu-Leu-OH	1717.0<br>(1718.1)	430.4 (4+)<br>573.7 (3+)<br>860.4 (2+)<br>**1717.6**	1.86<br>(1.05)
**24**<br>[ala^{8}]-LmC12-U2	H-arg-O2Oc-Leu-Gln-Lys-Asn-Val-Asn-Leu-ala-Lys-Tyr-Leu-Leu-OH	1717.0<br>(1718.1)	430.4 (4+)<br>573.7 (3+)<br>860.3 (2+)<br>**1717.6**	1.90<br>(1.06)
**25**<br>[lys^{9}]-LmC12-U2	H-arg-O2Oc-Leu-Gln-Lys-Asn-Val-Asn-Leu-Ala-lys-Tyr-Leu-Leu-OH	1717.0<br>(1718.1)	430.4 (4+)<br>573.7 (3+)<br>859.8 (2+)<br>**1717.6**	1.82<br>(1.04)
**26**<br>[tyr^{10}]-LmC12-U2	H-arg-O2Oc-Leu-Gln-Lys-Asn-Val-Asn-Leu-Ala-Lys-tyr-Leu-Leu-OH	1717.0<br>(1718.1)	430.6 (4+)<br>573.7 (3+)<br>859.8 (2+)<br>**1717.6**	1.90<br>(1.06)
**27**<br>[leu^{11}]-LmC12-U2	H-arg-O2Oc-Leu-Gln-Lys-Asn-Val-Asn-Leu-Ala-Lys-Tyr-leu-Leu-OH	1717.0<br>(1718.1)	430.3 (4+)<br>573.3 (3+)<br>859.8 (2+)<br>**1717.2**	1.87<br>(1.08)
**28**	H-arg-O2Oc-Leu-Gln-Lys-Asn-Val-Asn-Leu-Ala-Lys-	1717.0	430.6 (4+)	1.99

[leu^{12}]-LmC12-U2	Tyr-Leu-leu-OH	(1718.1)	573.7 (3+)<br>859.8 (2+)<br>**1717.6**	(1.10)
**Truncated peptides** (*C-ter LmHslU2*)				
**29**<br>LmC9-U2	H-arg-O2OC-Asn-Val-Asn-Leu-Ala-Lys-Tyr-Leu-Leu-OH	1347.8<br>(1348.6)	450.5 (3+)<br>675.0 (2+)<br>**1348.5**	2.25<br>(1.00)
**30**<br>LmC8-U2	H-arg-O2Oc-Val-Asn-Leu-Ala-Lys-Tyr-Leu-Leu-OH	1233.7<br>(1234.5)	412.4 (3+)<br>618.0 (2+)<br>1234.7 (1+)	1.98<br>(1.10)
**31**<br>LmC7-U2	H-arg-O2Oc-Asn-Leu-Ala-Lys-Tyr-Leu-Leu-OH	1134 .7<br>(1135.4)	379.3 (3+)<br>568.4 (2+)<br>1135.6 (1+)	1.88<br>(1.05)
**32**<br>Ac-LmC7-U2	Ac-Asn-Leu-Ala-Lys-Tyr-Leu-Leu-OH	875.5<br>(876.0)	438.7 (2+)<br>876.6 (1+)	2.09<br>(1.26)
**33**<br>LmC6-U2	H-arg-O2Oc-Leu-Ala-Lys-Tyr-Leu-Leu-OH	1020.6<br>(1021.2)	341.4 (3+)<br>511.4 (2+)<br>1021.7 (1+)	1.85<br>(1.03)
**34**<br>Ac-LmC6-U2	Ac-Leu-Ala-Lys-Tyr-Leu-Leu-OH	761.5<br>(761.9)	381.7 (2+)<br>762.5 (1+)	2.08<br>(1.27)
**35**<br>LmC4-U2	H-arg-O2Oc-Lys-Tyr-Leu-Leu-OH	836.5<br>(837.0)	419.4 (2+)<br>837.6 (1+)	1.54<br>(0.88)
**36**<br>Ac-LmC4-U2	Ac-Lys-Tyr-Leu-Leu-OH	577.3<br>(577.7)	289.8 (2+)<br>578.2 (1+)	1.67<br>(1.02)
**Cyclic peptides** (*C-ter LmHslU2*)				
**37**<br>cyclo[Asp^{4}, Lys^{8}]-LmC12-U2	H-arg-O2Oc-Leu-Gln-Lys-c[Asp-Val-Asn-Leu-Lys]-Lys-Tyr-Leu-Leu-OH	1757.1<br>(1758.1)	440.4 (4+)<br>586.8 (3+)<br>879.6 (2+)<br>**1757.5**	1.96<br>(1.01)
**38**<br>cyclo[asp^{4}, Lys^{8}]-LmC12-U2	H-arg-O2Oc-Leu-Gln-Lys-c[asp-Val-Asn-Leu-Lys]-Lys-Tyr-Leu-Leu-OH	1757.1<br>(1758.1)	440.5 (4+)<br>586.9 (3+)<br>879.7 (2+)<br>**1757.8**	1.92<br>(0.99)
**39**<br>cyclo[Asp^{4}, lys^{8}]-LmC12-U2	H-arg-O2Oc-Leu-Gln-Lys-c[Asp-Val-Asn-Leu-lys]-Lys-Tyr-Leu-Leu-OH	1757.1<br>(1758.1)	440.5 (4+)<br>586.8 (3+)<br>879.7 (2+)<br>**1757.6**	1.95<br>(1.17)
**40**<br>cyclo[asp^{4}, lys^{8}]-LmC12-U2	H-arg-O2Oc-Leu-Gln-Lys-c[asp-Val-Asn-Leu-lys]-Lys-Tyr-Leu-Leu-OH	1757.1<br>(1758.1)	440.4 (4+)<br>586.8 (3+)<br>880.1 (2+)<br>**1757.5**	1.99<br>(0.98)
**41**<br>cyclo[Asp^{4}, Orn^{8}]-LmC12-U2	H-arg-O2Oc-Leu-Gln-Lys-c[Asp-Val-Asn-Leu-Orn]-Lys-Tyr-Leu-Leu-OH	1743.1<br>(1744.1)	437.0 (4+)<br>582.2 (3+)<br>872.6 (1+)	2.18<br>(1.11)
**42**<br>cyclo[Asp^{4}, Dap^{8}]-LmC12-U2	H-arg-O2Oc-Leu-Gln-Lys-c[Asp-Val-Asn-Leu-Dap]-Lys-Tyr-Leu-Leu-OH	1715.1<br>(1716.1)	429.8 (4+)<br>572.8 (3+)<br>858.5 (2+)<br>**1715.2**	1.94<br>(0.98)
**43**<br>cyclo[Asp^{4}	H-arg-O2Oc-c[Asp-Val-Asn-Leu-Lys]-Lys-Tyr-Leu-Leu-OH	1387.8<br>(1388.7)	463.9 (3+)<br>695.2 (2+)	2.02<br>(1.00)

, Lys[8]]-<br>LmC9-U2			1388.8 (1+)	
**Scrambled LmC12-U2**				
**44**<br>Scr-<br>LmC12-U2	H-arg-O2Oc-Asn-Leu-Tyr-Lys-Leu-Val-Gln-Leu-Leu-Asn-Lys-Ala-OH	1717.0<br>(1718.1)	573.4 (3+)<br>860.0 (2+)	2.18<br>(1.22)

[a]Monoisotopic (and average) molecular weight. [b]ESI-MS: the molecular weight calculated from the observed multicharged species is indicated in bold. [c]Retention times measured by analytical RP-HPLC and LC-MS (in parentheses); 5 min gradient for analytical RP-HPLC, excepted [d]3 min gradient. Dap = Diaminopropionyl; Orn = Ornithine; O2Oc = $-NH(CH_2)_2O(CH_2)_2OCH_2CO-$.

Table S2. Peptide concentrations in CD experiments determined by measuring the absorbance at λ = 276 nm.

**Compounds**	**Structure**	**M + xTFA**	**O. D.**	**Conc. (µM)**
**10**	H-arg-O2Oc-Leu-Gln-Lys-Asn-Val-Asn-Leu-Ala-Lys-Tyr-Leu-Leu-OH	1717 + 456	0.331	228
**29**	H-arg-O2Oc-Asn-Val-Asn-Leu-Ala-Lys-Tyr-Leu-Leu-OH	1348 + 342	0.260	179
**30**	H-arg-O2Oc-Val-Asn-Leu-Ala-Lys-Tyr-Leu-Leu-OH	1233 + 342	0.296	204
**37**	H-arg-O2Oc-Leu-Gln-Lys-c[Asp-Val-Asn-Leu-Lys]-Lys-Tyr-Leu-Leu-OH	1757 + 456	0.315	217
**43**	H-arg-O2Oc-c[Asp-Val-Asn-Leu-Lys]-Lys-Tyr-Leu-Leu-OH	1388 + 342	0.240	165
